# MiRNA Expression in Patients with Gaucher Disease Treated with Enzyme Replacement Therapy

**DOI:** 10.3390/life11010002

**Published:** 2020-12-22

**Authors:** Łukasz Pawliński, Anna Polus, Ewa Tobór, Maria Sordyl, Marianna Kopka, Bogdan Solnica, Beata Kieć-Wilk

**Affiliations:** 1 Clinical Department of Metabolic Diseases and Diabetology, University Hospital in Krakow, 30-688 Kraków, Poland; upawlinski@gmail.com (Ł.P.); ewatobor@gmail.com (E.T.); piasek.mm@gmail.com (M.K.); 2 European Reference Network for Hereditary Metabolic Disorders (MetabERN), 31-501 Kraków, Poland; 3 Department of Clinical Biochemistry, Jagiellonian University Medical College, 30-688 Kraków, Poland; a.polus@uj.edu.pl (A.P.); maria.biela@uj.edu.pl (M.S.); mbsolnic@cyf-kr.edu.pl (B.S.); 4 Department of Metabolic Diseases and Diabetology, Jagiellonian University Medical College, 30-688 Kraków, Poland

**Keywords:** biomarkers, Gaucher disease, inflammation, miRNA

## Abstract

Aims: The aim of the work was to establish potential biomarkers or drug targets by analysing changes in miRNA concentration among patients with Gaucher disease (GD) compared to in healthy subjects. Methods: This study was an observational, cross-sectional analysis of 30 adult participants: 10 controls and 20 adults with GD type 1. Patients with GD type 1 were treated with enzyme replacement therapy (ERT) for at least two years. The control group was composed of healthy volunteers, unrelated to the patients, adjusted with age, sex and body mass index (BMI). The miRNA alteration between these groups was examined. After obtaining preliminary results on a group of six GD patients by the high-output method (TaqMan low-density array (TLDA)), potential miRNAs were selected for confirming the results by using the qRT-PCR method. With Diane Tools, we analysed miRNAs of which differential expression is most significant and their potential role in GD pathophysiology. We also determined the essential pathways these miRNAs are involved in. Results: 266 dysregulated miRNAs were found among 753 tested. Seventy-eight miRNAs were downregulated, and 188 were upregulated. Thirty miRNAs were significantly altered; all of them were upregulated. The analysis of pathways regulated by the selected miRNAs showed an effect on bone development, inflammation or regulation of axonal transmission in association with Parkinson’s disease. Conclusions: We revealed few miRNAs, like miR-26-5p, which are highly altered and fit the GD pathophysiological model, might be considered as novel biomarkers of disease progression but need further evaluation.

## 1. Introduction

Among lysosomal storage diseases, Gaucher disease (GD) is one of the most common. It is estimated that around 1 per 57,000 newborns is affected worldwide [1]. The disease’s phenotype is associated with a mutation in the lysosomal glucosylceramidase (*GBA*) gene; however, it differs remarkably even in patients with the same type of mutation [2,3,4]. Three types of GD are recognized upon central nervous system (CNS) involvement: non-neuronopathic (GD type 1: OMIM #230800), acute neuronopathic (GD type 2: OMIM #230900) and chronic neuronopathic (GD type 3: OMIM #231000). The most common form of GD, covering approximately 94% of cases, is type 1, which can manifest itself at any age [5]. It is characterised by haematological changes such as thrombocytopenia, anaemia and highly pronounced splenomegaly. Sometimes, hepatomegaly may coexist, and changes occurring in the skeletal system are also important [5]. Type 2 with a very severe course, regardless of the treatment attempts, leads to death around two years of age, while type 3, in addition to organ changes, is also associated with the involvement of the central nervous system, characterised by the presence of drug-resistant epilepsy; however, in this case, patients live to adulthood [5].

GD patients are also more susceptible to some diseases like heamatological neoplasms, Parkinson’s disease (PD) or central nervous involvement than the normal population [6]. 

One of the suspected reasons for that variability is an epigenetic mechanism that can modulate the course of the illness [7]. That is carried by, among others, microRNAs (miRNA), which are small noncoding RNA molecules found in almost all life forms and are responsible for RNA silencing and post-transcriptional regulation of gene expression. Therefore, to delve into it, we marked the expression of 753 miRNAs in GD patients, compared it to that in the control, healthy group, and for those miRNAs that were altered, we evaluated their potential role, compared with available data and analysed molecular pathways that they are involved in. All of the tested miRNAs are shown in the Appendix A (Table A1).

Currently, the possible role of organ-specific miRNAs in predicting the development of metabolic diseases such as diabetes and its complications or the development of complications in the course of lysosomal storage diseases (LSDs) is widely discussed [8,9]. Due to the stability of miRNAs in body fluids and distribution in specific tissues, it may turn these particles into a desirable biomarker of certain diseases [9]. Siebert, in his work on fibroblast cell lines from GD patients, showed that some miRNAs had a strong effect on the regulation of β-glucocerebrosidase (GCase) activity [10]. Two miRNAs (miR-195-5p and miR-16-5p) strongly stimulated *GBA1* gene expansion, while miR-127-5p, miR-19a-5p and miR-1262 inhibited the expression of SCARB2, a membrane receptor regulating GCase activity and availability [10]. MicroRNAs were shown to downregulate *GBA* and *GBAP1* (miR-22-3p) [11]. Some miRNAs like miR-let7b, miR-29b or miR-142 were associated with proinflammatory events [12]. Recently, Watson et al. described an unsuccessful ablation of miR-155, which was identified in knocked-down *gba1* zebrafish (in vivo GD model) as a proinflammatory master regulator [13]. However, the work on the role of miRNAs in GD only focused on the modifying role of this epigenetic mechanism in *GBA* gene expression [10]. It is worth remembering that most of these works concerned in vivo and in vitro observation, and we identified only one human observation [10].

Nevertheless, the big picture is still missing. Therefore, our work aimed to investigate changes in miRNAs concentration in patients with GD who were treated with enzyme replacement therapy (ERT) and to try finding new diagnostic markers or potential targets for treatment.

## 2. Material and Methods

This study was an observational, cross-sectional analysis of 30 adult participants: 20 adults with GD type 1 and 10 controls. First, samples from 8 random persons (4 from the GD group and 4 from the control one) were used for the TaqMan low-density array (TLDA), a high-throughput method for identifying alterations in miRNAs expression levels. Based on these preliminary results, potential miRNAs were selected for a confirmation on a whole group (30 persons) using the more precise qRT-PCR method. Patients with GD type 1 were treated in the Metabolic Diseases and Diabetes Department in the Jagiellonian University Hospital in Krakow, part of EU reference centres—MetabERN. The study included adult people with GD, treated in our centre, who agreed to participate in the study and signed up an informed consent. GD diagnosis was established on the positive genetic or/and enzymatic tests and, in two cases, additional histopathological examination. All these patients underwent ERT for at least two years. The exclusion factors for the analysis were as follows: neoplastic diseases, PD, autoimmune diseases, pregnancy, infection, fever or lack of patient’s consent to participate in the study. The control group was composed of healthy volunteers, not related to the patients, adjusted with age, sex and BMI.

All the participants underwent a detailed physical examination (age, weight, height, BMI, age and any disease history). Fasting venous blood was used for molecular biology tests. Molecular tests were performed at the Clinical Biochemistry Department of the Jagiellonian University. The clinical characteristic of groups is presented in Table 1.

## 3. The miRNA Analysis 

### 3.1. RNA Isolation 

The purification of total RNA from human whole blood was performed using the PAXgene Blood RNA Kit (Qiagen, Germantown, MD, USA), following the manufacturer’s protocol. The RNA quality was analysed using the Tapestation 2200 instrument (Agilent Technologies, Santa Clara, CA, USA) and quantified by spectrophotometry on the NanoDrop (Thermo Fisher Scientific, Wilmington, DE, USA). 

### 3.2. TLDA

The initial expression analysis of human microRNAs (753 unique assays) was performed by a high-throughput method using the TLDAs Panel v3.0 (Applied Biosystems, Foster City, CA, USA), as described by the manufacturer. Total RNA enriched with microRNAs was reverse-transcribed using stem-loop primers. In order to detect low abundant microRNAs, a preamplification step was performed. The preamplified product was loaded into a TLDA, and amplification signal detection was performed in the 7900 FAST real-time thermal cycler (ABI) (Thermo Fisher Scientific, Wilmington, DE, USA). Data were exported to the DataAssist software version 3.01 (Life Technologies, now Thermo Fisher Scientific, Waltham, MA, USA) and normalised using the small-nucleolar RNA U6snRNA. Mean relative quantity (RQ) was calculated, and microRNAs differentially expressed between groups were defined as those with a fold change of >1.5 and a *p*-value of <0.05. The differentially expressed miRNAs based on the TLDAs results were verified using microRNA assays (TaqMan Array Human MicroRNA Panel v3.0). 

### 3.3. qRT-PCR

Subsequently, to validate the microarray experiment, qRT-PCR was conducted on 20 patients with GD type 1 and 10 volunteers from the control group. Total RNA was reverse-transcribed using a specific stem-loop RT primer for each microRNA and the MultiScribeTM reverse transcriptase (Thermo Fisher Scientific, Wilmington, DE, USA). Then, diluted retro-transcription reaction (1:15) was mixed with the Universal PCR Master Mix, No AmpErase R UNG (2X), in the presence of individual PCR microRNAs. The PCR reactions were done in a GeneAmp R PCR System 9700 (Applied Biosystems, Foster City, CA, USA), using the following program: 95 °C for 10 min, 40 cycles of 95 °C for 15 s and 60 °C for 1 min. The relative expression of microRNAs was measured by qRT-PCR using the comparative Ct (∆∆CT) method. A two-tailed Student’s t-test comparing the 2(–ΔCt) values of the two groups was performed, and the Benjamini–Hochberg-adjusted *p*-value was calculated. The U6 snoRNA was used as an internal control for data normalisation. 

### 3.4. Pathway Enrichment

MicroRNA targets were predicted using a microRNA target prediction program: DIANA-microT (http://diana.imis.athena-innovation.gr/DianaTools/index.php?r=microT_CDS/index). Biological pathways of predicted target-genes microRNAs were identified using Kyoto Encyclopedia of Genes and Genomes (KEGG) pathway analysis (http://www.genome.jp/kegg/pathway.html) and mirPath v.3 (http://snf-515788.vm.okeanos.grnet.gr/).

Briefly, the DIANA-mirPath utilised predicted miRNA targets provided by the miRNA-gene interactions derived from experimentally supported DIANA-TarBase v.7.0 (Department of Electrical and Computer Engineering, 382 21 Volos, Greece) or microT-CDS target prediction, if there were no hits for DIANA-TarBase. The KEGG pathways union was used to identify the main pathways targeted by predicted genes (discovered miRNA targets).

### 3.5. Statistical Analysis of Patients’ Baseline Characteristics 

For the analysis of the characteristics of the study groups, IBM SPSS Statistic Software was used. The Shapiro–Wilk test was performed to check the normality. Differences between groups were assessed using the T-test or U-test for normally and non-normally distributed continuous variables, respectively, and by the chi-square or Fischer’s exact test for categorical variables. Continuous variables are presented as mean ± SD or median (interquartile range). Statistical significance was set at *p* < 0.05.

## 4. Results

The analysed groups: control and GD patients were comparable in gender distribution, age and anthropometric parameters. To unify patients’ group as much as possible, all of the patients with GD type 1 were treated with ERT.

Using the TLDA method, we were able to perform an accurate quantitation of 753 microRNAs in the study group. U6 comparison data were used for normalization. Alterations in miRNAs expression were determined in 266 cases, among which 78 were downregulated and 188 were upregulated. Eventually, among all tested miRNAs, we identified 30 miRNAs, of which the expression significantly differed between GD patients and our control data (Table 2). Interestingly, all these miRNAs were upregulated in relation to the control group.

The most prominent alteration in the GD group included miR-26b-5p, miR-31-5p, miR-29a-3p, miR-454-3p, miR-660-5p and miR-148a-3p. The miRNAs were mapped for molecular pathways to identify those, which could be specifically modified in GD patients (Table 3). Among pathways, we chose the most noticeable ones according to the *p*-value, the proportion of miRNAs to controlled genes and actual knowledge about the pathophysiology of the lysosomal storage disease.

**Figure 1:**
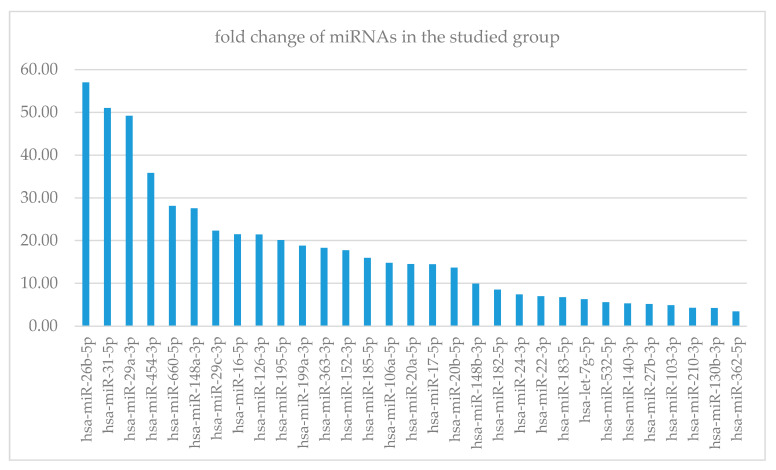
Fold change of miRNA concentration between GD group and control. X-axis—miRNAs, Y-axis—fold change.

The analysis using a heatmap (Figure A1) showed a strong relationship between the TGF-beta signal transmission pathway and miR-106a-5p. In addition, a significant association was found between pathways of fatty acid synthesis and metabolism, with miR-16-5p and miR-195-5p. Interestingly, these miRNAs played also a significant role in the regulation of the Akt-signalling pathway, focal adhesion or lysine degradation pathways. Figure 1 Additionally, we noted an important impact of differentially expressed miRNA on a very complex pathway of extracellular matrix (ECM)–receptor interaction, namely miR-29a-3p or miR-let-7g-5p.

## 5. Discussion

### 5.1. Comparison to Available Data

The biological purpose of miRNAs is related to their conservative function, common for many lifeforms, to control groups of genes by muting them when needed. They influence biochemical pathways on many levels to create a complex and tangled network of dependencies and controlling apparatus.

Nowadays, research to recognise miRNA in-depth role in the ability to regulate genes expression is blooming. As for GD, only a few miRNAs analyses have been described so far. In 2014, Siebert reported data for two miRNAs: 195-5p and 16-5p concentration changes in GD and linked them to the regulation (increase) of GCase activity [10]. Our data showed the upregulation of these two. In the presence of GCase insufficiency, the results suggested a secondary hyperconcentration of regulators and may confirm in vivo the importance of miR-195-5p and miR-16-5p in GCase secretion.

### 5.2. GD and Malignancies

miR-195-5p was also recently found to be downregulated in a broad spectrum of solid cancers. It was described as a cancer suppressor [14], which mediates its effect by complex and still vogue pathways. Similarly, miR-16-5p was described as a protective factor in breast carcinoma [15], and again, we showed significant upregulation of this miRNA.

Highly elevated levels of miR-195-5p and 16-5p may provide a protective role in GD in terms of solid tumours [16,17,18]. Until now, GD patients were found to be more susceptible primarily for haematologic neoplasms [19]. However, the data of breast cancer in GD are inconsistent. In 2015, European Registry did not report any incidence of breast cancer; neither did Shiran in 1993 [19,20]. In 2009, Taddei reported nine cases of breast cancer, with a risk ratio of 1.84 [21]. Therefore, these observations require further studies.

For miR-148b-3p, the connection between high concentration and poor survival in breast cancer was established [22]. Our data showed a 27.5-fold increase in this miRNA in the study group, but again, there are no available studies that investigated the mentioned issue.

Few papers described an increased number of cases with hepatocellular carcinoma (HCC) [23]. Zhao in 2017 revealed that the expression of miR-31-5p was significantly upregulated in HCC tissues [24]. This information is consistent with our analysis showing highly altered miR-31-5p that was more than 50 times higher than in the comparison group. In our tested groups, there was no HCC incidence. The question remains: is miR-31-5p just a marker for HCC or does it present the properties for the highly sensitive biomarker of the liver lesion which could reveal the destruction of hepatic cells, even when classical tests such as alanine aminotransferase (ALT) and aspartate aminotransferase (AST) are within a normal range? 

All the patients in the study group were on ERT for at least two years. This treatment could influence the concentrations of many miRNAs and change the course of the disease, including complication occurrence. However, the data from European Registry [20] did not reveal differences in cancer incidence between patients ever treated with ERT and those never treated with ERT, but still, the impact of ERT on miRNAs, which are associated with malignancies, is limited as the data are missing. There are also no data on the dependence of the miRNA concentration on the ERT presence. 

### 5.3. Other Highly Upregulated miRNA

The most prominent upregulated miRNA was found to be miR-26b-5p, which was linked with *TRPS1* gene, which is responsible for regulating genes involved in the growth of bone and cartilage. Therefore *TRPS1* controls chondrocyte proliferation and differentiation. MiR-26b-5p controls also bone morphogenetic protein 2 (BMP-2) that affects osteoblasts and regulates proliferation, differentiation and apoptosis [25]. In patients with GD, bone involvement is one of the main symptoms and consists of distortion of the vertebra and distension of long bones, known as Erlenmeyer flask bone deformity, with infiltration of bone marrow. It has been demonstrated that miR-26b-5p with a 66-fold increase of expression in our study group is a potential biomarker of bone involvement.

### 5.4. miRNA Pathways Analysis

To put altered miRNAs into the bigger picture, we used the DIANA-mirPath v3 tool for analysing miRNA-predicted targets with KEGG pathways. Nineteen pathways were significantly altered. Those, most noticeable influenced and possibly important in GD pathophysiology are “TGF-beta signalling pathway”, “focal adhesion”, “FoxO signalling pathway”, “axon guidance” and “ECM–receptor interaction”.

The TGF-beta signalling pathway is found in numerous human disorders. As it controls the growth inhibitors, the alteration of its activity is observed in cancers [26], which are more frequent in GD. In addition, TGF-beta stimulates ECM deposition that may lead to fibrosis and scarring [27]. The higher prevalence of liver fibrosis in GD was also shown [28].

The focal adhesion pathway focuses on the regulation of call–matrix interaction. The central point of that pathway—focal adhesion kinase (FAK) was roughly studied in terms of cancer progression [29,30,31]. Likewise, FAK was found to promote proinflammatory gene expression via TNF-α or IL-1β [32].

The FoxO signalling pathway, on the other hand, is involved in oxidation stress and may play an important role in neurodegenerative disease like Parkinson’s [33,34]. 

The axon guidance pathways involve, among others, miR-20a-5p, 106a-5p, 27b-3p or 182-5p as regulators, which in our analysis showed significant overexpression. Among these, 182-5p was found to interact with cofilin-1 (*CFL1*) and further axon growth [34]. As axon guidance is responsible for controlling neurons morphogenesis and signalling processes [35], its malfunction may contribute to neuropathy in GD patients. 

These data confirmed the previously described role of inflammatory processes and the disrupted pathways of cell migration and interaction with the ECM, that consists of a mixture of structural and macromolecules and serves an important role not only in tissue morphogenesis but also in the regulation of cell and tissue structure and function.

### 5.5. miRNA and PD 

Since the relationship between PD and GD was revealed, many studies were trying to establish the underlying pathophysiology. It was shown that patients with PD had a decreased level of GCase in the brain tissue. Moreover, even carries of the single *GBA* gene mutation present tendency towards PD [36]. This finding showed that GD patients should be more susceptible to the occurrence of PD, which was confirmed in [36]. Research on the expression of genes related to neuroinflammation and synaptic plasticity are ongoing [12]. MiR-26b and miR-106a-5p acting through *HSPA8* [37], as well as miR-7 and miRNA-153, are found to increase the concentration of alpha-synuclein [38], the primary accumulation material in PD. We noted a 14-fold increase of miR-106-5p in our study group, which is consistent with the known data.

## 6. Conclusions

GD patients are still in need of new biomarkers and therapeutic targets that can help evaluate disease progression or treatment effectiveness. We highlighted some miRNA compounds that showed remarkable alteration in those patients and might be potential biomarkers. However, due to scarce data on GD, these findings need further studies.

## Figures and Tables

**Table 1:** Clinical characteristics of study participants with GD (*n* = 20) and the control group (*n* = 10).

Characteristic	GD Patients	Control	*p*-Value
Sex (F/M)	60/40%	50/50%	0.53
Age (years)	23–73 (median: 36.6)	24–60 (median: 37.5)	0.43
First symptoms (age)	1–55 (median: 16)	N/A	N/A
Weight (kg)	39–71 (median: 63)	59–89 (median: 71)	0.53
Height (cm)	138–175 (median: 162)	164–181 (median: 169)	0.73
BMI (kg/m^2^)	20.5–25.5 (median: 24.81)	20.9–29 (median: 23.59)	0.20
Splenectomy	2/18 (10%)	0/10 (0%)	0.54

**Table 2:** 30 significantly altered microRNA level in GD patient’s group vs control; U6 was used as a reference.

miRNA	Gaucher vs. U6	
	Fold Change	*p*
hsa-let-7g-5p	6.2815	0.0323
hsa-miR-103-3p	4.8908	0.0053
hsa-miR-106a-5p	14.7874	0.0082
hsa-miR-126-3p	21.3974	0.0362
hsa-miR-130b-3p	4.2382	0.0303
hsa-miR-140-3p	5.2985	0.0446
hsa-miR-148a-3p	27.5481	0.0158
hsa-miR-148b-3p	9.9113	0.0076
hsa-miR-152-3p	17.7294	0.0241
hsa-miR-155-5p	11.8767	0.0016
hsa-miR-16-5p	21.4519	0.0343
hsa-miR-17-5p	14.4325	0.0084
hsa-miR-182-5p	8.5183	0.0001
hsa-miR-183-5p	6.7224	0.0059
hsa-miR-185-5p	15.9617	0.0206
hsa-miR-195-5p	20.1085	0.0083
hsa-miR-199a-3p	18.8214	0.0462
hsa-miR-20a-5p	14.4807	0.0079
hsa-miR-20b-5p	13.6756	0.0317
hsa-miR-210-3p	4.2888	0.0299
hsa-miR-22-3p	6.9943	0.0246
hsa-miR-24-3p	7.4002	0.0091
hsa-miR-26b-5p	57.02	0.0351
hsa-miR-27b-3p	5.172	0.0081
hsa-miR-29a-3p	49.1923	0.0239
hsa-miR-31-5p	51.0322	0.0004
hsa-miR-363-3p	18.2858	0.0267
hsa-miR-454-3p	35.8331	0.0242
hsa-miR-532-5p	5.6011	0.0102
hsa-miR-660-5p	28.1258	0.0357

**Table 3:** KEGG pathway analysis of miRNA target genes, significantly altered with *p* < 0.05.

#KEGG Pathway	*p*-Value	#Genes	#miRNAs
Fatty acid biosynthesis	<0.0001	4	2
Prion diseases	<0.0001	1	4
ECM-receptor interaction	<0.0001	45	9
Glioma	<0.0001	35	11
Signaling pathways regulating pluripotency of stem cells	<0.0001	69	8
Proteoglycans in cancer	<0.0001	97	11
TGF-beta signaling pathway	<0.0001	43	9
FoxO signaling pathway	<0.0001	49	9
Metabolism of xenobiotics by cytochrome P450	<0.0001	13	5
Pathways in cancer	0.0001048971	118	6
Prostate cancer	0.000309422	33	4
Amoebiasis	0.0004413348	23	2
Mucin type O-Glycan biosynthesis	0.001082663	12	4
PI3K-Akt signaling pathway	0.001397357	89	7
Focal adhesion	0.001524581	70	4
Renal cell carcinoma	0.002187101	19	5
Melanoma	0.002618092	26	5
Fatty acid metabolism	0.005693764	7	2
Axon guidance	0.01611344	43	4

**Figure A1:**
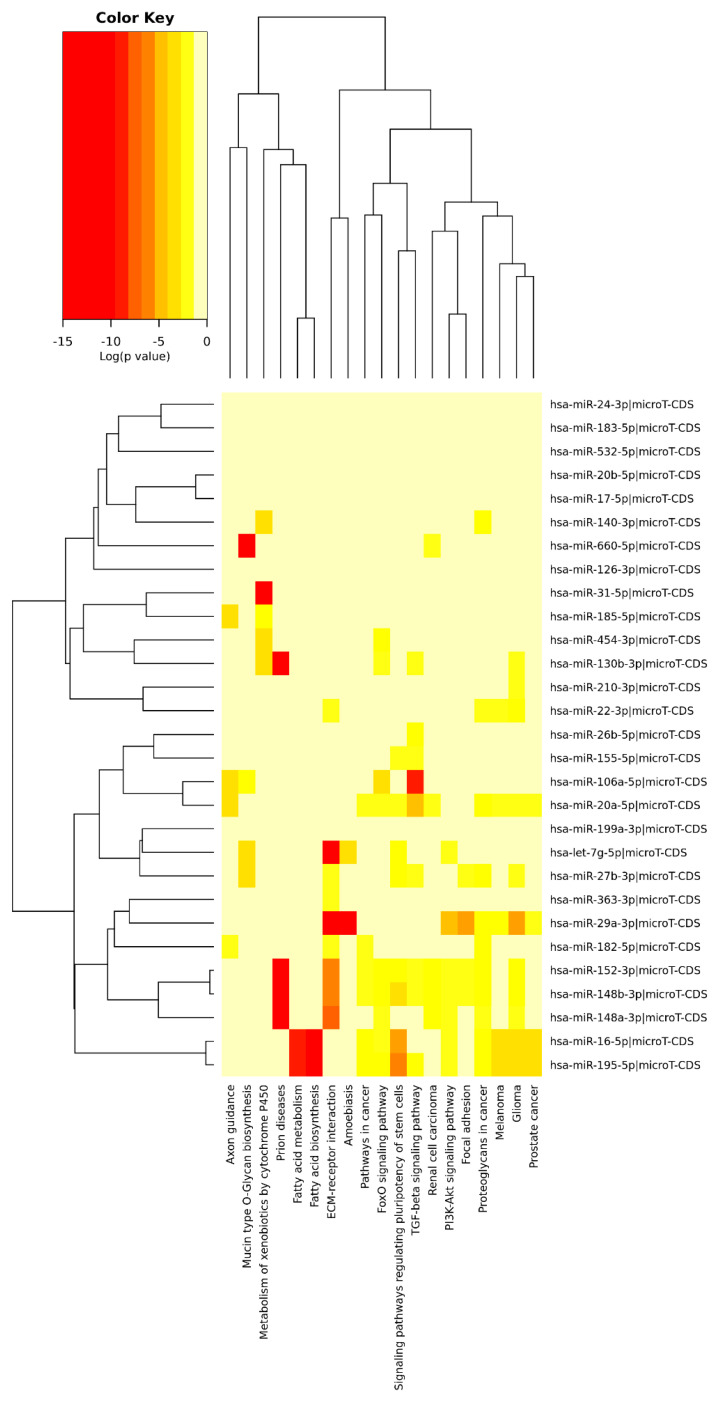
Heatmap presenting miRNA impact in specific pathways.

## Appendix A

**Table A1:** List of all of the tested miRNAs. For 266 altered miRNAs: fold change, *p*-value and SD data are presented.

miRNA	Fold Change	*p*-Value	SD
hsa-miR-152-000475	18.1382	0.0003	1.956398
hsa-miR-454-002323	38.5767	0.0004	0.887427
mmu-miR-140-001187	32.7431	0.0022	0.796695
hsa-miR-532-001518	6.7361	0.0068	1.858579
hsa-miR-148b-000471	11.4268	0.0084	0.930063
hsa-miR-17-002308	16.9061	0.0088	1.105731
hsa-miR-148a-000470	32.9918	0.0089	1.748115
mmu-miR-93-001090	5.7093	0.0089	0.81462
hsa-miR-106a-002169	17.3487	0.0095	1.104199
hsa-miR-20a-000580	17.0211	0.0097	1.115662
hsa-miR-324-5p-000539	26.0859	0.01	0.863699
hsa-miR-16-000391	24.6779	0.0102	1.212765
hsa-miR-660-001515	32.8325	0.0105	1.537621
hsa-miR-126-002228	24.5953	0.0106	1.214868
hsa-miR-26b-000407	66.9029	0.011	0.894591
hsa-miR-29c-000587	22.6399	0.0111	0.729712
hsa-miR-140-3p-002234	6.0836	0.0112	1.477748
hsa-miR-30b-000602	4.2752	0.0156	1.025196
hsa-miR-106b-000442	34.8823	0.0158	1.020234
hsa-miR-146b-001097	28.6668	0.0171	0.745407
mmu-miR-451-001141	22.3568	0.0173	1.281826
hsa-miR-212-000515	4.014	0.0191	1.005979
hsa-miR-31-002279	61.5757	0.0225	3.805273
hsa-miR-182-002334	10.9175	0.0269	1.014663
hsa-miR-20b-001014	15.102	0.0281	0.968618
hsa-miR-185-002271	19.9198	0.0291	1.583043
hsa-miR-27a-000408	22.6143	0.0302	1.593832
hsa-miR-22-000398	8.934	0.0317	1.16643
hsa-miR-26a-000405	19.104	0.0328	1.213354
mmu-miR-374-5p-001319	10.4528	0.0329	1.260503
hsa-miR-128a-002216	15.2816	0.033	2.046559
hsa-miR-18a-002422	40.5059	0.0367	1.138078
hsa-miR-210-000512	5.1833	0.0379	1.053872
hsa-miR-194-000493	30.5951	0.0388	1.389726
hsa-miR-192-000491	31.244	0.0389	0.902125
hsa-miR-191-002299	5.5793	0.0401	1.600253
hsa-miR-28-000411	19.4532	0.0405	0.901813
hsa-miR-146a-000468	30.847	0.0429	1.382615
hsa-miR-130b-000456	5.3197	0.0433	1.578003
hsa-miR-184-000485	5.5476	0.0444	0.8337
hsa-miR-362-001273	3.8332	0.0449	0.995367
hsa-miR-130a-000454	37.3076	0.0469	1.578769
hsa-miR-500-002428	2.8525	0.047	1.113499
hsa-miR-195-000494	26.5732	0.0487	1.167158
hsa-miR-199a-3p-002304	23.1246	0.0534	1.014663
hsa-miR-502-3p-002083	3.3767	0.0548	0.812816
hsa-miR-155-002623	9.7213	0.0577	1.37917
hsa-miR-590-5p-001984	324.1959	0.0578	0.642024
hsa-miR-24-000402	9.9502	0.0581	1.384917
hsa-miR-374-000563	73.6336	0.0587	0.699017
hsa-miR-339-3p-002184	13.3932	0.0609	1.538581
hsa-miR-425-5p-001516	3.957	0.061	1.162153
hsa-miR-27b-000409	6.8449	0.0625	1.167805
hsa-miR-25-000403	7.1621	0.063	1.363864
hsa-miR-29a-002112	62.4411	0.0656	1.976845
hsa-miR-345-002186	7.4086	0.066	1.380509
hsa-miR-183-002269	9.2849	0.0686	0.889644
hsa-miR-331-000545	3.9872	0.0687	1.152766
hsa-miR-103-000439	6.6069	0.0699	1.220862
hsa-miR-101-002253	19.0521	0.0701	0.592054
hsa-miR-30c-000419	3.1992	0.0723	1.025552
hsa-miR-133a-002246	9.0033	0.0726	1.228076
hsa-miR-223-002295	16.2644	0.0772	1.533045
hsa-miR-744-002324	2.6774	0.0798	1.38857
hsa-miR-221-000524	2.9226	0.0807	1.312939
hsa-miR-363-001271	25.8516	0.0846	1.616082
hsa-miR-376c-002122	33.9427	0.0911	0.474342
hsa-miR-139-5p-002289	7.0336	0.0913	1.214447
hsa-miR-132-000457	3.8428	0.096	1.014592
hsa-miR-301-000528	64.8911	0.0976	0.509057
hsa-miR-181c-000482	4.708	0.1	1.0808
hsa-let-7g-002282	8.7654	0.1014	1.107341
hsa-let-7f-000382	9.0417	0.1016	0.968148
hsa-miR-361-000554	18.4177	0.1029	2.978323
hsa-miR-21-000397	48.0159	0.103	0.939588
hsa-miR-200c-002300	3.3312	0.1051	1.512568
hsa-miR-186-002285	16.6037	0.1108	0.786326
hsa-miR-27a#-002445	7.3705	0.1117	0.965802
hsa-miR-502-001109	2.6428	0.1137	0.975355
hsa-miR-501-001047	2.5867	0.1185	1.056212
hsa-miR-222-002276	11.368	0.1195	1.742912
hsa-miR-342-5p-002147	3.5973	0.1248	1.323263
hsa-miR-126#-000451	40.9923	0.126	0.935753
hsa-miR-19b-000396	91.4227	0.1261	0.75054
hsa-miR-19a-000395	190.411	0.1311	0.747321
hsa-let-7e-002406	4.4222	0.1334	1.317315
hsa-miR-28-3p-002446	2.6331	0.1365	0.962861
hsa-miR-539-001286	10.361	0.1371	0.73037
hsa-miR-1233-002768	0.1814	0.1381	1.095964
hsa-miR-376a-000565	6.0198	0.1459	0.517776
hsa-miR-629-002436	4.9459	0.1487	1.014592
hsa-miR-324-3p-002161	3.153	0.1501	1.014592
hsa-miR-1255B-002801	9.2195	0.1519	1.384534
hsa-miR-335-000546	57.1738	0.1536	0.403013
hsa-miR-142-3p-000464	140.5412	0.1621	0.664435
hsa-miR-181a-000480	3.2078	0.1621	1.395227
hsa-miR-1227-002769	0.1024	0.1633	0.966204
hsa-let-7a-000377	3.2189	0.1692	1.31003
hsa-let-7d-002283	3.2535	0.1707	1.318228
hsa-miR-204-000508	13.0355	0.1763	1.520136
hsa-miR-10a-000387	5.052	0.1827	0.691164
hsa-miR-1260-002896	0.1645	0.1841	1.057165
hsa-miR-628-3p-002434	5.0633	0.1875	1.168615
hsa-miR-365-001020	2.3993	0.19	1.761739
hsa-miR-744#-002325	4.6279	0.1909	2.144439
hsa-miR-1290-002863	0.1228	0.1913	0.690733
hsa-miR-370-002275	5.138	0.1967	0.96788
hsa-miR-520c-3p-002400	0.2261	0.197	1.062601
hsa-miR-1238-002927	0.0698	0.2007	1.014592
hsa-miR-26b#-002444	6.0929	0.2032	1.40211
rno-miR-7#-001338	79.9503	0.2054	3.010285
hsa-miR-1282-002803	0.1537	0.2059	2.198623
hsa-miR-1285-002822	6.123	0.206	1.266722
hsa-miR-1249-002868	0.1254	0.2069	0.976979
hsa-miR-190b-002263	6.6002	0.209	1.199389
hsa-miR-193a-5p-002281	2.3905	0.2092	1.38934
hsa-miR-151-5P-002642	5.9735	0.2108	1.25475
hsa-miR-211-000514	3.3212	0.2137	2.352509
hsa-miR-486-001278	0.1804	0.2163	1.319325
hsa-miR-505-002089	3.0798	0.2207	1.078406
U6 snRNA-001973	1.6133	0.2224	0.81169
hsa-miR-223#-002098	13.2203	0.225	1.208402
hsa-miR-1224-3P-002752	0.0546	0.2252	1.175195
hsa-miR-26a-1#-002443	11.4173	0.2262	1.014592
hsa-miR-655-001612	2.2436	0.2283	1.230803
hsa-miR-15b-000390	3.0261	0.2297	1.222556
hsa-miR-618-001593	2.3227	0.2301	0.922167
hsa-miR-100-000437	5.4736	0.2319	0.585037
hsa-miR-1236-002761	0.0953	0.2334	1.026049
hsa-miR-196b-002215	9.1066	0.2345	1.099845
hsa-miR-139-3p-002313	2.3313	0.2359	1.557573
hsa-miR-197-000497	0.1018	0.2361	1.170318
hsa-miR-425#-002302	3.2533	0.2364	1.391461
hsa-miR-99a-000435	3.9774	0.2402	0.919231
hsa-miR-494-002365	2.5905	0.2431	1.178213
hsa-miR-148b#-002160	21.1813	0.2489	0.967343
hsa-miR-149-002255	0.2668	0.25	1.386839
hsa-miR-340#-002259	16.3674	0.2523	1.117907
hsa-miR-15b#-002173	114.3628	0.2532	1.582275
hsa-miR-454#-001996	6.9324	0.2536	0.891743
hsa-miR-144#-002148	179.9935	0.255	1.021295
hsa-miR-296-000527	0.23	0.2556	1.161589
hsa-miR-584-001624	3.8238	0.2574	0.922039
hsa-miR-550-002410	3.6444	0.265	1.583152
hsa-miR-30d-000420	5.3738	0.2702	1.231059
hsa-miR-29b-2#-002166	4.3903	0.2736	1.164088
hsa-miR-328-000543	0.1902	0.2799	0.890693
mmu-miR-134-001186	5.3946	0.2847	0.644924
hsa-miR-625-002431	2.1806	0.2892	1.320697
hsa-miR-378-002243	11.6798	0.2899	1.533789
hsa-miR-192#-002272	4.5328	0.2919	0.890693
hsa-miR-22#-002301	9.6977	0.2946	0.896205
hsa-miR-1253-002894	0.1835	0.2951	1.154926
hsa-miR-935-002178	0.0435	0.2961	1.020729
hsa-miR-30a-5p-000417	8.8831	0.2968	1.22731
hsa-let-7c-000379	2.681	0.2996	1.06157
hsa-miR-629-001562	0.4522	0.302	1.308125
hsa-miR-574-3p-002349	0.3825	0.3115	0.928903
hsa-miR-342-3p-002260	1.8466	0.3137	1.487201
hsa-miR-769-5p-001998	3.0669	0.3139	0.922231
hsa-miR-1183-002841	0.2419	0.3154	1.205056
rno-miR-29c#-001818	25.3746	0.321	1.22748
hsa-miR-1178-002777	2.7188	0.3247	1.084252
hsa-miR-1274A-002883	4.1151	0.3266	0.916877
hsa-miR-483-3p-002339	0.0888	0.3271	1.396001
hsa-miR-942-002187	5.4029	0.3287	1.014592
hsa-miR-361-3p-002116	3.0612	0.3291	1.195654
hsa-miR-335#-002185	7.5039	0.3302	1.063264
hsa-miR-652-002352	2.3486	0.3309	2.001248
hsa-miR-151-3p-002254	14.1994	0.3345	1.027401
hsa-miR-30e-3p-000422	14.3748	0.3347	1.0208
hsa-miR-766-001986	0.4831	0.3352	1.162234
hsa-miR-19b-1#-002425	10.9526	0.336	1.023137
hsa-miR-636-002088	0.398	0.3372	1.014592
hsa-miR-616-001589	4.8155	0.3429	1.223403
hsa-miR-30a-3p-000416	11.0253	0.3433	0.960262
hsa-miR-375-000564	3.193	0.3442	0.95482
dme-miR-7-000268	13.7859	0.3474	0.683352
hsa-miR-213-000516	8.8128	0.3514	1.946524
RNU44-001094	0.6987	0.3528	1.078181
hsa-miR-202-002363	0.2041	0.353	0.975491
hsa-miR-668-001992	0.4798	0.3633	0.511569
hsa-miR-589-001543	2.1132	0.3735	0.811071
hsa-miR-92a-000431	0.4571	0.3768	1.014592
hsa-miR-1274B-002884	3.8258	0.3852	1.028969
hsa-miR-382-000572	3.4752	0.3988	0.281596
hsa-miR-206-000510	0.1562	0.407	1.024273
hsa-miR-885-5p-002296	0.4147	0.4116	0.890569
hsa-miR-433-001028	0.2961	0.4183	0.394829
hsa-miR-1254-002818	0.5042	0.4233	0.967612
hsa-let-7b-002619	1.6905	0.4289	1.39175
hsa-miR-886-5p-002193	1.9418	0.4298	0.921784
hsa-miR-642-001592	0.4673	0.4354	0.966271
hsa-miR-1208-002880	0.1609	0.4369	0.724069
hsa-miR-564-001531	0.1813	0.4433	1.924922
hsa-miR-485-3p-001277	0.466	0.4481	0.722816
hsa-miR-1201-002781	2.7108	0.4534	2.211308
hsa-miR-1248-002870	0.4995	0.4543	1.096496
hsa-miR-1271-002779	2.5278	0.4558	1.014592
hsa-miR-518d-001159	0.2429	0.4564	1.010872
hsa-miR-191#-002678	2.4958	0.4632	1.393391
hsa-miR-505#-002087	0.5571	0.4665	1.014663
hsa-miR-424#-002309	1.9373	0.473	2.100889
hsa-miR-484-001821	1.4584	0.4773	1.327121
hsa-miR-939-002182	0.3419	0.4916	0.858149
hsa-miR-34b-002102	1.4845	0.4924	1.288328
hsa-miR-645-001597	0.1907	0.4934	0.938026
hsa-miR-1300-002902	0.6216	0.4978	1.225525
hsa-miR-25#-002442	0.5651	0.5019	1.001804
mmu-miR-491-001630	1.5858	0.5058	1.014592
hsa-miR-504-002084	0.1667	0.5178	1.20882
hsa-miR-605-001568	0.4642	0.533	1.336259
hsa-miR-486-3p-002093	0.1683	0.5418	1.014874
hsa-miR-625#-002432	0.6167	0.5519	1.020305
hsa-miR-409-3p-002332	1.2673	0.5525	0.693323
hsa-miR-320-002277	1.5302	0.5542	0.925112
hsa-miR-720-002895	0.5745	0.5651	0.776361
hsa-miR-1303-002792	0.265	0.5794	1.087262
hsa-miR-99b-000436	0.6432	0.5833	1.373636
hsa-let-7e#-002407	0.51	0.5843	1.084778
hsa-miR-423-5p-002340	0.5315	0.5853	0.89199
hsa-miR-320B-002844	0.5947	0.5889	0.967746
hsa-miR-671-3p-002322	0.6999	0.5962	1.382711
hsa-miR-338-5P-002658	0.6849	0.6055	1.014592
hsa-miR-326-000542	1.6869	0.6102	1.116203
hsa-miR-616-002414	1.6343	0.6257	1.015366
hsa-miR-483-5p-002338	0.3713	0.6263	0.741439
hsa-miR-601-001558	0.4504	0.642	0.742159
hsa-miR-18a#-002423	0.7197	0.6483	1.391268
hsa-miR-181a-2#-002317	1.4607	0.6512	1.404932
hsa-miR-331-5p-002233	0.43	0.6549	0.884724
hsa-miR-130b#-002114	1.4513	0.6589	1.20072
hsa-miR-129#-002298	0.7193	0.6649	1.014522
hsa-miR-589-002409	1.5415	0.6777	0.962128
hsa-miR-543-002376	0.7598	0.6838	0.972116
hsa-miR-127-000452	0.7153	0.6839	0.714547
RNU48-001006	1.1272	0.6986	1.259106
hsa-miR-1270-002807	0.6817	0.7017	1.219509
hsa-miR-422a-002297	1.338	0.7159	0.966204
hsa-miR-93#-002139	1.2568	0.7245	1.161428
hsa-miR-654-001611	0.5941	0.7281	1.353411
hsa-miR-329-001101	0.5892	0.7404	0.888658
hsa-miR-363#-001283	0.7238	0.7439	1.09013
hsa-miR-532-3p-002355	0.7953	0.7439	1.014663
hsa-miR-875-5p-002203	1.4066	0.7492	0.504876
hsa-miR-125a-5p-002198	0.811	0.7623	1.374112
hsa-miR-664-002897	0.8292	0.7715	1.378787
hsa-miR-145-002278	1.1687	0.7947	1.385878
hsa-miR-150-000473	1.1539	0.8032	1.696075
hsa-miR-1180-002847	0.8312	0.8039	1.014522
hsa-miR-432-001026	0.821	0.8238	0.87043
hsa-miR-106b#-002380	1.1768	0.8323	0.965735
hsa-miR-146b-3p-002361	1.2251	0.855	1.25562
hsa-miR-125b-000449	1.1266	0.8657	0.929612
hsa-miR-378-000567	1.1219	0.876	1.168777
hsa-miR-17#-002421	1.1894	0.8838	0.814508
hsa-miR-183#-002270	1.1154	0.884	1.014944
hsa-miR-323-3p-002227	0.8646	0.8974	1.334038
hsa-miR-571-001613	0.8796	0.9149	1.038931
hsa-miR-193b-002367	1.0645	0.9241	1.387897
hsa-miR-501-3p-002435	0.9202	0.9298	2.597277
hsa-miR-550-001544	1.0424	0.9392	1.389051
hsa-miR-1275-002840	0.9498	0.9519	1.065256
hsa-miR-330-000544	0.9717	0.9652	1.383862
hsa-miR-339-5p-002257	0.9757	0.9707	1.168858
hsa-miR-148a#-002134	1.04	0.9717	0.916305
mmu-miR-495-001663	0.9631	0.9763	0.684537
hsa-miR-941-002183	0.9924	0.9897	1.16546
hsa-miR-20a#-002437	0.9973	0.9984	15.10234
ath-miR159a-000338			
hsa-let-7a#-002307			
hsa-let-7b#-002404			
hsa-let-7c#-002405			
hsa-let-7f-1#-002417			
hsa-let-7f-2#-002418			
hsa-let-7g#-002118	1.4648		
hsa-let-7i#-002172	107.2379		
hsa-miR-100#-002142			
hsa-miR-1-002222			
hsa-miR-101#-002143			
hsa-miR-105#-002168			
hsa-miR-105-002167			
hsa-miR-106a#-002170			
hsa-miR-107-000443			
hsa-miR-10a#-002288			
hsa-miR-10b#-002315			
hsa-miR-10b-002218			
hsa-miR-1179-002776			
hsa-miR-1182-002830			
hsa-miR-1184-002842			
hsa-miR-1197-002810			
hsa-miR-1200-002829			
hsa-miR-1203-002877			
hsa-miR-1204-002872			
hsa-miR-1205-002778			
hsa-miR-1206-002878			
hsa-miR-122#-002130			
hsa-miR-122-002245	0.5039		
hsa-miR-1225-3P-002766			
hsa-miR-1226#-002758			
hsa-miR-1228#-002763			
hsa-miR-124#-002197			
hsa-miR-1243-002854			
hsa-miR-1244-002791	3.1723		
hsa-miR-1245-002823			
hsa-miR-1247-002893	0.0859		
hsa-miR-1250-002887			
hsa-miR-1251-002820			
hsa-miR-1252-002860			
hsa-miR-1255A-002805			
hsa-miR-1256-002850			
hsa-miR-1257-002910			
hsa-miR-1259-002796			
hsa-miR-125a-3p-002199	0.531		
hsa-miR-125b-1#-002378			
hsa-miR-125b-2#-002158			
hsa-miR-1262-002852			
hsa-miR-1263-002784			
hsa-miR-1264-002799			
hsa-miR-1265-002790			
hsa-miR-1267-002885			
hsa-miR-1269-002789			
hsa-miR-1272-002845			
hsa-miR-127-5p-002229			
hsa-miR-1276-002843			
hsa-miR-1278-002851			
hsa-miR-1283-002890			
hsa-miR-1284-002903			
hsa-miR-1286-002773			
hsa-miR-1288-002832			
hsa-miR-1289-002871			
hsa-miR-129-000590			
hsa-miR-1291-002838	6.764		
hsa-miR-1292-002824			
hsa-miR-1293-002905			
hsa-miR-1294-002785			
hsa-miR-1296-002908	5.0843		
hsa-miR-1298-002861			
hsa-miR-1301-002827			
hsa-miR-1302-002901			
hsa-miR-1304-002874			
hsa-miR-1305-002867			
hsa-miR-130a#-002131			
hsa-miR-132#-002132			
hsa-miR-1324-002815			
hsa-miR-133b-002247	88.5947		
hsa-miR-135a-000460			
hsa-miR-135b#-002159			
hsa-miR-135b-002261	0.1506		
hsa-miR-136#-002100			
hsa-miR-136-000592			
hsa-miR-138-002284			
hsa-miR-138-2#-002144			
hsa-miR-141#-002145			
hsa-miR-141-000463			
hsa-miR-142-5p-002248			
hsa-miR-143#-002146			
hsa-miR-143-002249			
hsa-miR-144-002676			
hsa-miR-145#-002149			
hsa-miR-146a#-002163			
hsa-miR-147-000469			
hsa-miR-147b-002262			
hsa-miR-149#-002164			
hsa-miR-154#-000478			
hsa-miR-154-000477			
hsa-miR-155#-002287			
hsa-miR-15a#-002419	107.6923		
hsa-miR-15a-000389			
hsa-miR-16-1#-002420	218.8413		
hsa-miR-16-2#-002171			
hsa-miR-181c#-002333			
hsa-miR-182#-000483			
hsa-miR-1825-002907			
hsa-miR-1826-002873			
hsa-miR-185#-002104			
hsa-miR-186#-002105			
hsa-miR-188-3p-002106			
hsa-miR-18b#-002310			
hsa-miR-18b-002217	211.9679		
hsa-miR-190-000489			
hsa-miR-193a-3p-002250			
hsa-miR-193b#-002366	0.2068		
hsa-miR-194#-002379	0.1097		
hsa-miR-195#-002107			
hsa-miR-196a#-002336			
hsa-miR-198-002273			
hsa-miR-199a-000498			
hsa-miR-199b-000500			
hsa-miR-19a#-002424			
hsa-miR-200a#-001011			
hsa-miR-200a-000502			
hsa-miR-200b#-002274			
hsa-miR-200b-002251	21.2685		
hsa-miR-200c#-002286			
hsa-miR-202#-002362	9.7132		
hsa-miR-203-000507			
hsa-miR-205-000509	10.5591		
hsa-miR-208-000511			
hsa-miR-208b-002290			
hsa-miR-20b#-002311			
hsa-miR-21#-002438	11.3163		
hsa-miR-214#-002293			
hsa-miR-214-002306	0.0964		
hsa-miR-215-000518			
hsa-miR-216a-002220			
hsa-miR-216b-002326			
hsa-miR-217-002337			
hsa-miR-218-000521	170.283		
hsa-miR-218-1#-002094			
hsa-miR-218-2#-002294			
hsa-miR-219-000522			
hsa-miR-219-1-3p-002095	30.3606		
hsa-miR-219-2-3p-002390			
hsa-miR-220-000523			
hsa-miR-220b-002206			
hsa-miR-220c-002211			
hsa-miR-221#-002096			
hsa-miR-222#-002097	43.7736		
hsa-miR-224-002099			
hsa-miR-23a#-002439			
hsa-miR-23a-000399	38.5634		
hsa-miR-23b#-002126			
hsa-miR-23b-000400	0.082		
hsa-miR-24-1#-002440			
hsa-miR-24-2#-002441			
hsa-miR-26a-2#-002115			
hsa-miR-27b#-002174			
hsa-miR-296-3p-002101			
hsa-miR-298-002190			
hsa-miR-299-3p-001015			
hsa-miR-299-5p-000600			
hsa-miR-29a#-002447			
hsa-miR-29b-000413			
hsa-miR-29b-1#-002165			
hsa-miR-301b-002392			
hsa-miR-302a#-002381			
hsa-miR-302a-000529			
hsa-miR-302b#-002119			
hsa-miR-302b-000531			
hsa-miR-302c#-000534			
hsa-miR-302c-000533			
hsa-miR-302d#-002120			
hsa-miR-302d-000535			
hsa-miR-30b#-002129			
hsa-miR-30c-1#-002108			
hsa-miR-30c-2#-002110			
hsa-miR-30d#-002305			
hsa-miR-31#-002113	11.1358		
hsa-miR-32#-002111			
hsa-miR-32-002109			
hsa-miR-325-000540			
hsa-miR-330-5p-002230			
hsa-miR-337-3p-002157	0.881		
hsa-miR-337-5p-002156			
hsa-miR-338-3p-002252	0.8708		
hsa-miR-33a#-002136			
hsa-miR-33a-002135			
hsa-miR-33b-002085			
hsa-miR-340-002258	26.6065		
hsa-miR-346-000553			
hsa-miR-34a#-002316			
hsa-miR-34a-000426			
hsa-miR-34b-000427			
hsa-miR-34c-000428			
hsa-miR-362-3p-002117	107.0275		
hsa-miR-367#-002121			
hsa-miR-367-000555			
hsa-miR-369-3p-000557			
hsa-miR-369-5p-001021			
hsa-miR-371-3p-002124			
hsa-miR-372-000560			
hsa-miR-373-000561			
hsa-miR-374a#-002125			
hsa-miR-374b#-002391			
hsa-miR-376a#-002127			
hsa-miR-376b-001102			
hsa-miR-377#-002128	0.8371		
hsa-miR-377-000566			
hsa-miR-380-3p-000569			
hsa-miR-380-5p-000570			
hsa-miR-381-000571			
hsa-miR-383-000573			
hsa-miR-384-000574			
hsa-miR-409-5p-002331			
hsa-miR-410-001274			
hsa-miR-411#-002238			
hsa-miR-411-001610			
hsa-miR-412-001023			
hsa-miR-424-000604			
hsa-miR-429-001024			
hsa-miR-431#-002312			
hsa-miR-431-001979			
hsa-miR-432#-001027	1.8383		
hsa-miR-448-001029			
hsa-miR-449-001030	125.8155		
hsa-miR-449b-001608			
hsa-miR-450a-002303			
hsa-miR-450b-3p-002208			
hsa-miR-450b-5p-002207			
hsa-miR-452#-002330			
hsa-miR-452-002329			
hsa-miR-453-002318			
hsa-miR-455-001280			
hsa-miR-455-3p-002244			
hsa-miR-485-5p-001036			
hsa-miR-487a-001279	0.0523		
hsa-miR-487b-001285	94.345		
hsa-miR-488-001106			
hsa-miR-488-002357			
hsa-miR-489-002358			
hsa-miR-490-001037			
hsa-miR-491-3p-002360			
hsa-miR-492-001039			
hsa-miR-493-002364			
hsa-miR-497#-002368			
hsa-miR-497-001043			
hsa-miR-499-3p-002427			
hsa-miR-500-001046	3.8468		
hsa-miR-503-001048			
hsa-miR-506-001050	23.0067		
hsa-miR-507-001051			
hsa-miR-508-001052	0.631		
hsa-miR-508-5p-002092			
hsa-miR-509-3-5p-002155			
hsa-miR-509-5p-002235			
hsa-miR-510-002241			
hsa-miR-511-001111			
hsa-miR-512-3p-001823			
hsa-miR-512-5p-001145			
hsa-miR-513-5p-002090			
hsa-miR-513B-002757			
hsa-miR-513C-002756			
hsa-miR-515-3p-002369			
hsa-miR-515-5p-001112			
hsa-miR-516-3p-001149	0.2851		
hsa-miR-516a-5p-002416			
hsa-miR-516b-001150			
hsa-miR-517#-001113			
hsa-miR-517a-002402			
hsa-miR-517b-001152			
hsa-miR-517c-001153			
hsa-miR-518a-3p-002397			
hsa-miR-518a-5p-002396			
hsa-miR-518b-001156			
hsa-miR-518c#-001158			
hsa-miR-518c-002401			
hsa-miR-518d-5p-002389			
hsa-miR-518e#-002371			
hsa-miR-518e-002395			
hsa-miR-518f#-002387			
hsa-miR-518f-002388			
hsa-miR-519a-002415			
hsa-miR-519b-3p-002384			
hsa-miR-519c-001163			
hsa-miR-519d-002403			
hsa-miR-519e#-001166			
hsa-miR-519e-002370			
hsa-miR-520a#-001168			
hsa-miR-520a-001167			
hsa-miR-520b-001116			
hsa-miR-520D-3P-002743			
hsa-miR-520d-5p-002393			
hsa-miR-520e-001119			
hsa-miR-520f-001120			
hsa-miR-520g-001121			
hsa-miR-520h-001170			
hsa-miR-521-001122			
hsa-miR-522-002413			
hsa-miR-523-002386			
hsa-miR-524-001173			
hsa-miR-524-5p-001982			
hsa-miR-525-001174			
hsa-miR-525-3p-002385			
hsa-miR-526b-002382			
hsa-miR-541#-002200			
hsa-miR-541-002201			
hsa-miR-542-3p-001284			
hsa-miR-542-5p-002240			
hsa-miR-544-002265			
hsa-miR-545#-002266	0.4262		
hsa-miR-545-002267			
hsa-miR-548a-001538	0.0416		
hsa-miR-548a-5p-002412			
hsa-miR-548b-001541			
hsa-miR-548b-5p-002408			
hsa-miR-548c-001590	5.3989		
hsa-miR-548c-5p-002429	38.7768		
hsa-miR-548d-001605	29.9772		
hsa-miR-548d-5p-002237			
hsa-miR-548E-002881			
hsa-miR-548G-002879			
hsa-miR-548H-002816			
hsa-miR-548I-002909			
hsa-miR-548J-002783	1.8774		
hsa-miR-548K-002819			
hsa-miR-548L-002904			
hsa-miR-548M-002775			
hsa-miR-548N-002888			
hsa-miR-548P-002798			
hsa-miR-549-001511			
hsa-miR-551a-001519	1.4235		
hsa-miR-551b#-002346			
hsa-miR-551b-001535			
hsa-miR-552-001520			
hsa-miR-553-001521			
hsa-miR-554-001522			
hsa-miR-555-001523			
hsa-miR-556-3p-002345			
hsa-miR-556-5p-002344			
hsa-miR-557-001525			
hsa-miR-558-001526			
hsa-miR-559-001527			
hsa-miR-561-001528			
hsa-miR-562-001529			
hsa-miR-563-001530			
hsa-miR-566-001533			
hsa-miR-567-001534			
hsa-miR-569-001536			
hsa-miR-570-002347			
hsa-miR-572-001614	0.437		
hsa-miR-573-001615			
hsa-miR-575-001617			
hsa-miR-576-3p-002351	3.0672		
hsa-miR-576-5p-002350			
hsa-miR-577-002675			
hsa-miR-578-001619			
hsa-miR-579-002398	130.8115		
hsa-miR-580-001621			
hsa-miR-581-001622			
hsa-miR-582-3p-002399			
hsa-miR-582-5p-001983			
hsa-miR-583-001623			
hsa-miR-585-001625			
hsa-miR-586-001539	0.0471		
hsa-miR-587-001540			
hsa-miR-588-001542			
hsa-miR-590-3P-002677			
hsa-miR-591-001545			
hsa-miR-592-001546			
hsa-miR-593-001547			
hsa-miR-593-002411			
hsa-miR-595-001987			
hsa-miR-596-001550			
hsa-miR-597-001551			
hsa-miR-598-001988	155.6581		
hsa-miR-599-001554			
hsa-miR-600-001556			
hsa-miR-603-001566			
hsa-miR-604-001567			
hsa-miR-606-001569			
hsa-miR-607-001570			
hsa-miR-608-001571			
hsa-miR-609-001573			
hsa-miR-613-001586			
hsa-miR-614-001587			
hsa-miR-615-5p-002353			
hsa-miR-617-001591			
hsa-miR-620-002672			
hsa-miR-621-001598			
hsa-miR-622-001553			
hsa-miR-623-001555			
hsa-miR-624-001557	0.9465		
hsa-miR-624-002430			
hsa-miR-626-001559			
hsa-miR-627-001560	228.4357		
hsa-miR-628-5p-002433	271.071		
hsa-miR-630-001563			
hsa-miR-631-001564			
hsa-miR-633-001574			
hsa-miR-634-001576			
hsa-miR-635-001578			
hsa-miR-637-001581			
hsa-miR-638-001582	0.746		
hsa-miR-639-001583	0.4233		
hsa-miR-640-001584			
hsa-miR-641-001585			
hsa-miR-643-001594			
hsa-miR-644-001596			
hsa-miR-646-001599			
hsa-miR-647-001600			
hsa-miR-648-001601			
hsa-miR-649-001602			
hsa-miR-650-001603			
hsa-miR-651-001604			
hsa-miR-653-002292			
hsa-miR-654-3p-002239			
hsa-miR-656-001510			
hsa-miR-657-001512			
hsa-miR-658-001513			
hsa-miR-659-001514			
hsa-miR-661-001606	2.7873		
hsa-miR-662-001607			
hsa-miR-663B-002857	0.1863		
hsa-miR-665-002681			
hsa-miR-672-002327			
hsa-miR-674-002021			
hsa-miR-675-002005			
hsa-miR-708#-002342			
hsa-miR-708-002341	0.4321		
hsa-miR-7-2#-002314	26.3744		
hsa-miR-758-001990	0.2387		
hsa-miR-765-002643			
hsa-miR-767-3p-001995			
hsa-miR-767-5p-001993			
hsa-miR-769-3p-002003	2.3061		
hsa-miR-770-5p-002002			
hsa-miR-802-002004			
hsa-miR-871-002354			
hsa-miR-872-002264			
hsa-miR-873-002356			
hsa-miR-874-002268	7.9572		
hsa-miR-875-3p-002204			
hsa-miR-876-3p-002225			
hsa-miR-876-5p-002205			
hsa-miR-885-3p-002372			
hsa-miR-886-3p-002194	1.2122		
hsa-miR-887-002374			
hsa-miR-888#-002213			
hsa-miR-888-002212			
hsa-miR-889-002202			
hsa-miR-890-002209			
hsa-miR-891a-002191	4.6329		
hsa-miR-891b-002210			
hsa-miR-892a-002195			
hsa-miR-892b-002214			
hsa-miR-9#-002231			
hsa-miR-9-000583			
hsa-miR-920-002150			
hsa-miR-921-002151			
hsa-miR-922-002152			
hsa-miR-924-002154			
hsa-miR-92a-1#-002137			
hsa-miR-92a-2#-002138			
hsa-miR-92b#-002343			
hsa-miR-933-002176			
hsa-miR-934-002177			
hsa-miR-936-002179			
hsa-miR-937-002180			
hsa-miR-938-002181			
hsa-miR-943-002188			
hsa-miR-944-002189			
hsa-miR-95-000433			
hsa-miR-96#-002140			
hsa-miR-98-000577			
hsa-miR-99a#-002141	0.0374		
hsa-miR-99b#-002196	0.0378		
mmu-let-7d#-001178			
mmu-miR-124a-001182			
mmu-miR-129-3p-001184	0.2208		
mmu-miR-137-001129			
mmu-miR-153-001191			
mmu-miR-187-001193			
mmu-miR-379-001138			
mmu-miR-496-001953			
mmu-miR-499-001352			
